# Supplementary figures and images for: Reactive oxygen species prevent lysosome coalescence during PIKfyve inhibition
Source: PLoS One. 2021 Nov 23;16(11):e0259313. doi: 10.1371/journal.pone.0259313 (PMC8610251; doi:10.1371/journal.pone.0259313)

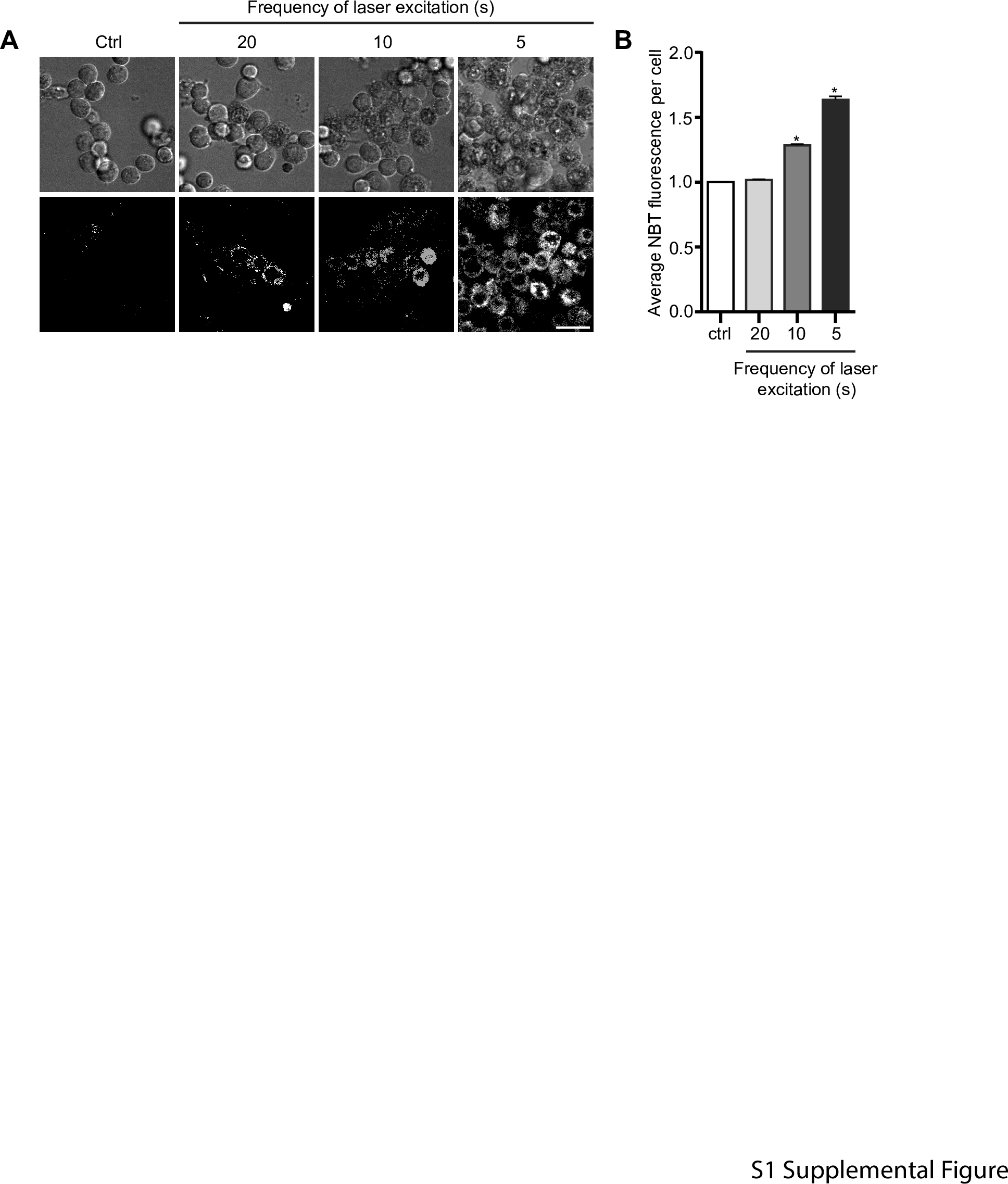

Supplement: S1 Fig — (A) RAW cells were loaded with Alexa546-conjugated dextran to label lysosomes and incubated with 1 mg/mL NBT for 30 min in dark. Cells were then exposed to the red laser to excite Alexa546-conjugated dextran every 20 s or 10 s or 5 s, or not exposed to the red laser (ctrl). The NBT fluorescence was then detected using far-red channel. Scale bar: 20 μm. (B) Quantification of mean NBT fluorescence per cell. Data represent ± SEM from three independent experiments, with 20–35 cells assessed per treatment condition per experiment. One-way ANOVA and Tukey’s post-hoc test was used, where * indicates statistical significance between indicated conditions (p<0.05). (TIF) [file pone.0259313.s001.tif]

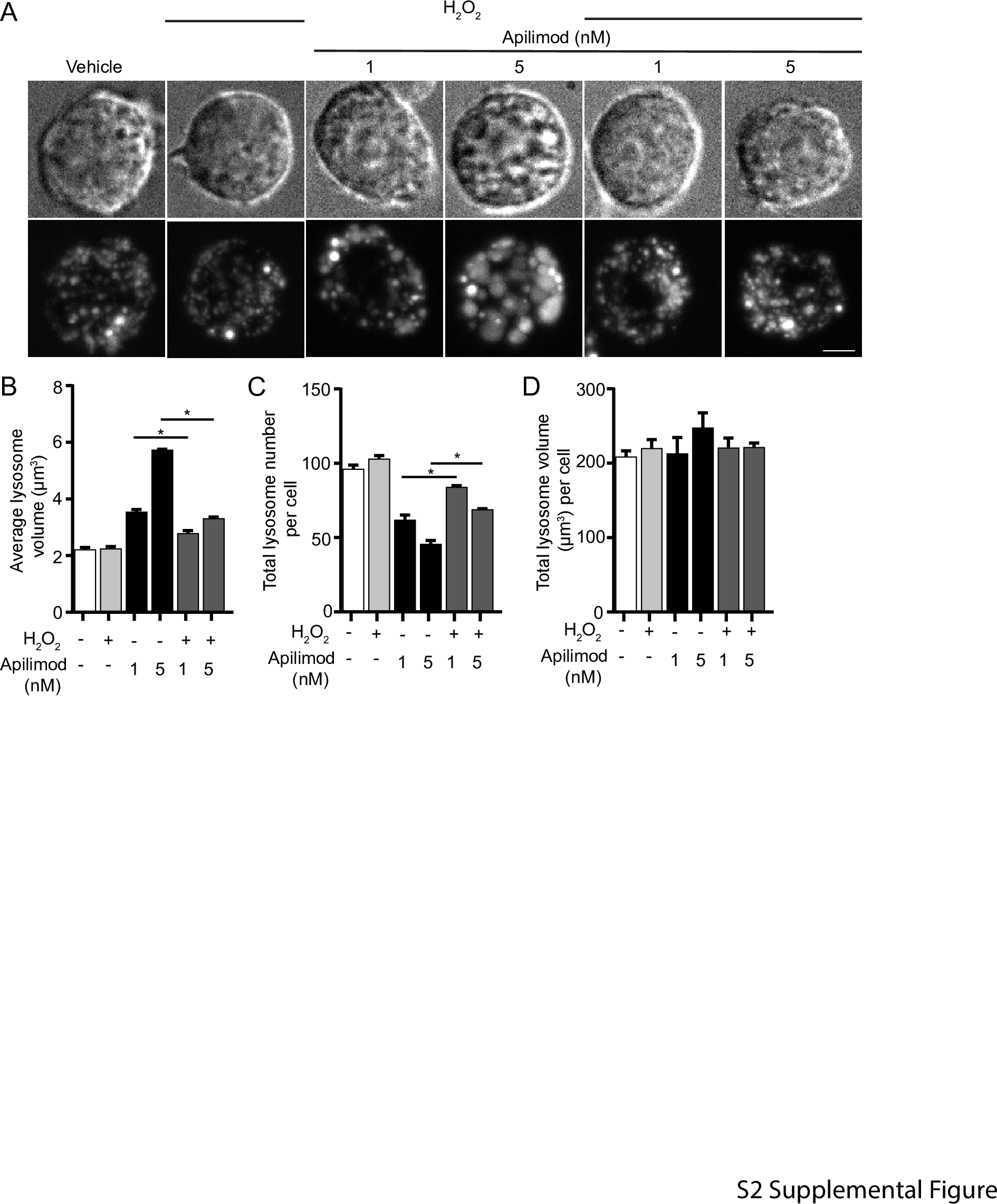

Supplement: S2 Fig — (A) RAW cells were pre-labelled with Lucifer yellow and exposed to either vehicle, 100 μM H2O2 for 40 min in presence or absence of 1 nM or 5 nM apilimod 40 min. Scale bar: 5 μm. (B-D) Quantification of individual lysosome volume (B), lysosome number per cell (C), and total lysosome volume per cell (D). Data are illustrated as mean ± SEM from three independent experiments, with 25–30 cell assessed per condition per experiment. One-way ANOVA and Tukey’s post-hoc test was used, where * indicates P<0.05 for the indicated conditions. (TIF) [file pone.0259313.s002.tif]

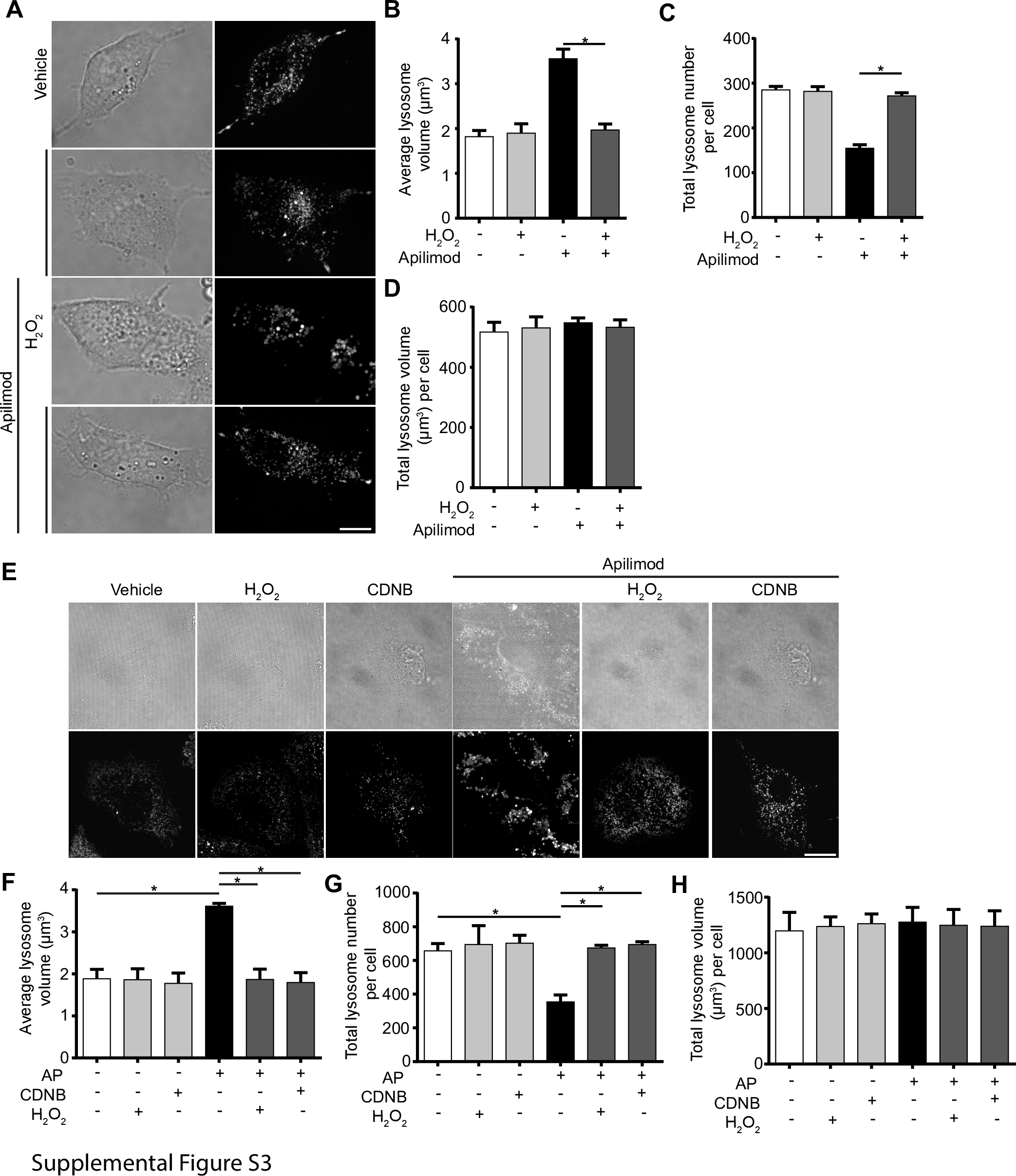

Supplement: S3 Fig — (A) HeLa cells pre-labelled with Lucifer yellow and exposed to vehicle or 100 nM apilimod 40 min, or with 1 mM H2O2 in the presence or absence of 100 nM apilimod for 40 min. Scale bar: 10 μm. (B-D) Quantification of individual lysosome volume per lysosome (B), lysosome number per cell (C), and total lysosome volume per cell (D). (E) RPE cells pre-labelled with Lucifer yellow and exposed to vehicle, or 1 mM H2O2, or 10 μM CDNB, in presence or absence of 200 nM apilimod 40 min. Scale bar: 20 μm. (F-H) Quantification of individual lysosome volume (F), lysosome number per cell (G), and sum lysosome volume per cell (H). For (B-D) and (F-H), data are represented as mean ± SEM. from three independent experiments, with 25–30 cells assessed for (B-D) and 15–20 cells assessed for (F-H) per treatment condition per experiment. One-way ANOVA and Tukey’s post-hoc test used with *P<0.05 compared to indicated control conditions. (TIF) [file pone.0259313.s003.tif]

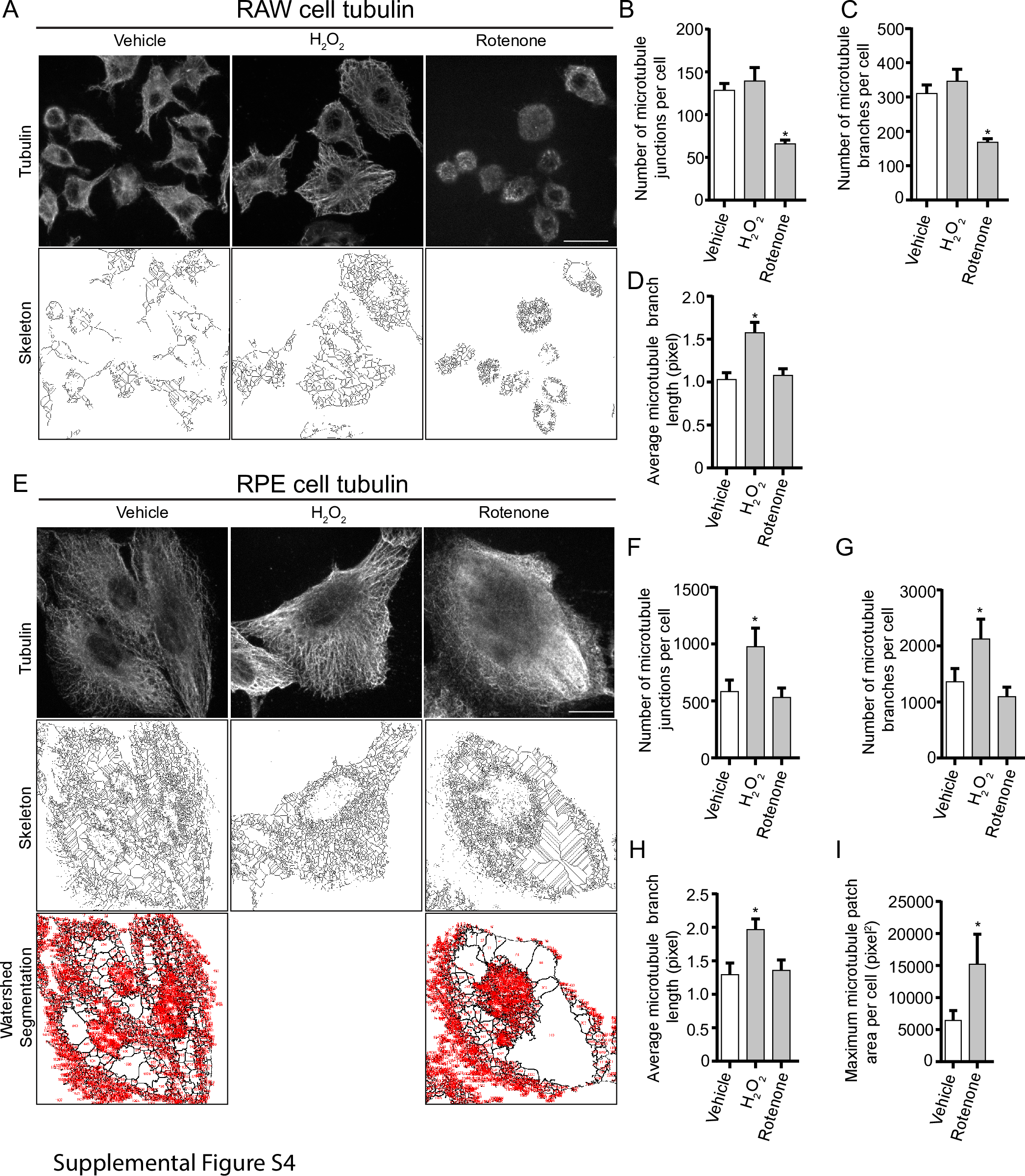

Supplement: S4 Fig — Single z-focal plane immunofluorescence micrographs of RAW cells (A) or RPE cells (E) treated with vehicle, H2O2 or rotenone. After treatment with ROS agents, cells were fixed and immunostained with anti-α-tubulin antibodies. Cells were analyzed for their microtubule morphology using the ImageJ “skeleton” plugin, converting images into binary “skeleton” micrographs. Quantification of number of microtubule junctions per cell, number of microtubule branches per cell and average microtubule branch length for RAW cells (B-D) and RPE cells (F-H). RPE cells were also analyzed for maximum microtubule patch area per cell (I) through ImageJ using binary filter and watershed segmentation. Data are represented as mean ± SD from 5 different fields of view for RAW cells or 10 different fields of view for RPE cell, with 50–70 cells assessed per treatment condition for RAW cells (A-D) and 15–20 cells assessed per treatment condition for RPE cells (E-H). One-way ANOVA and Tukey’s post-hoc test used for B-D and F-H, where * indicates statistically significant difference between control conditions (P<0.05). Scale bar: 20 μm (A, E). (TIF) [file pone.0259313.s004.tif]

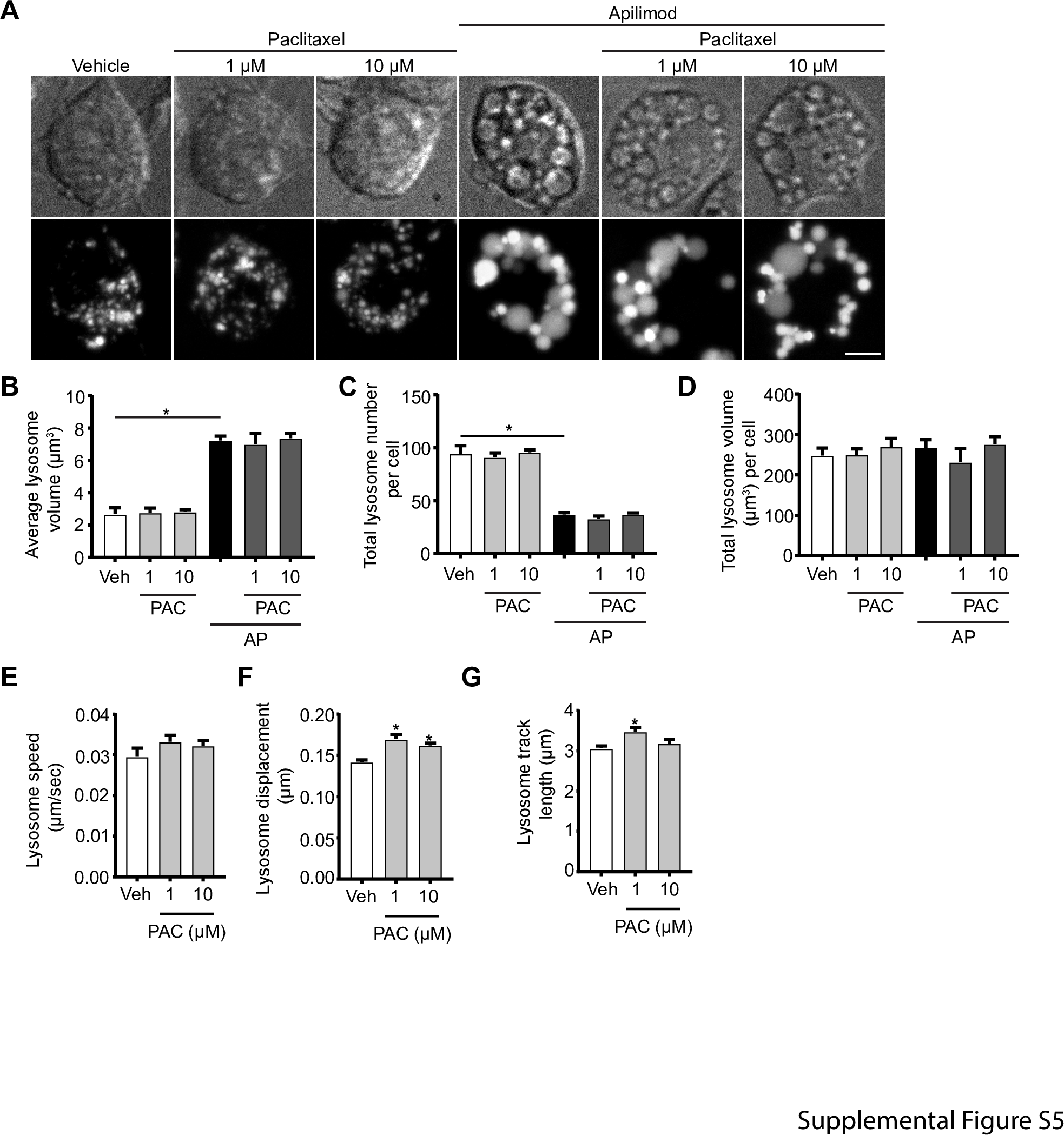

Supplement: S5 Fig — (A) RAW cells pre-labelled with Lucifer yellow were exposed to either vehicle, or 1 μM or 10 μM paclitaxel for 60 min in presence or absence of 20 nM apilimod for the remaining 40 min. Scale bar: 5 μm. (B-D) Quantification of individual lysosome volume (B), lysosome number per cell (C), and total lysosome volume per cell (D). Data are represented as mean ± s.e.m. from three independent experiments, with 25–30 cell assessed for (B-D) per treatment condition per experiment. (E-G) RAW cells pre-labelled with Lucifer yellow were exposed to vehicle or 1 μM or 10 μM paclitaxel 60 min. Live cell spinning disc confocal microscopy was performed at single z-focal plane once every 4 sec for 3 min. Quantification of lysosome speed (E), lysosome displacement (F), and lysosome track length (G) are shown. Data are represented as mean ± s.d. from three independent experiments. One-way ANOVA and Tukey’s post-hoc tests were used, where * indicates P<0.05 between experimental and control conditions. Data is based on movies like those represented by S14–S16 Movies. (TIF) [file pone.0259313.s005.tif]

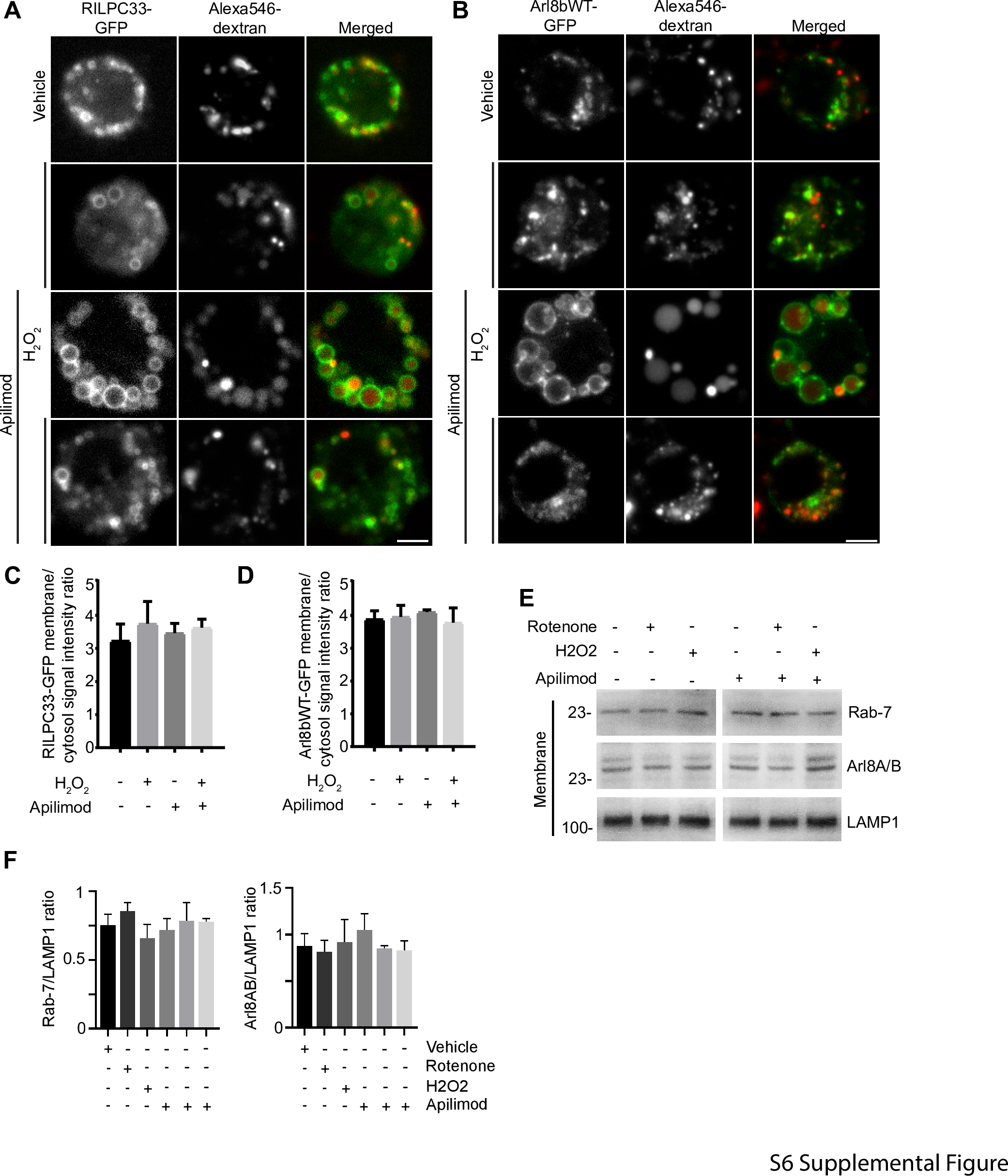

Supplement: S6 Fig — RAW cells expressing RILPC33-GFP (A), or Arl8bWT-GFP (B), exposed to vehicle in absence or presence of 20 nM apilimod 40 min, or 1 mM H2O2 40 min in presence or absence of 20 nM apilimod 40 min. Scale bar: 5 μm. (C-D) Quantification of membrane associated fluorescence intensity of RILPC33-GFP (C) from (A) or Arl8bWT-GFP (D) from (B), normalized to cytosol fluorescence intensity. Data represent mean ± SEM from three independent experiments, with 15–20 cell assessed per treatment condition per experiment. One-way ANOVA and Tukey’s post-hoc test used for C-D with *P<0.05 compared to indicated control conditions. (E) A representative Western blot of membrane fractions from RAW macrophages treated with vehicle, rotenone, or H2O2 with or without apilimod. Blots were probed with antibodies against Rab7, Arl8a/b, and LAMP1, the latter used to benchmark membrane levels. (F) Relative levels of Arl8ab/b or Rab7 as a ratio to LAMP1 band intensity. Data are shown as mean + standard deviation from n = 3 independent experiments. (TIF) [file pone.0259313.s006.tif]

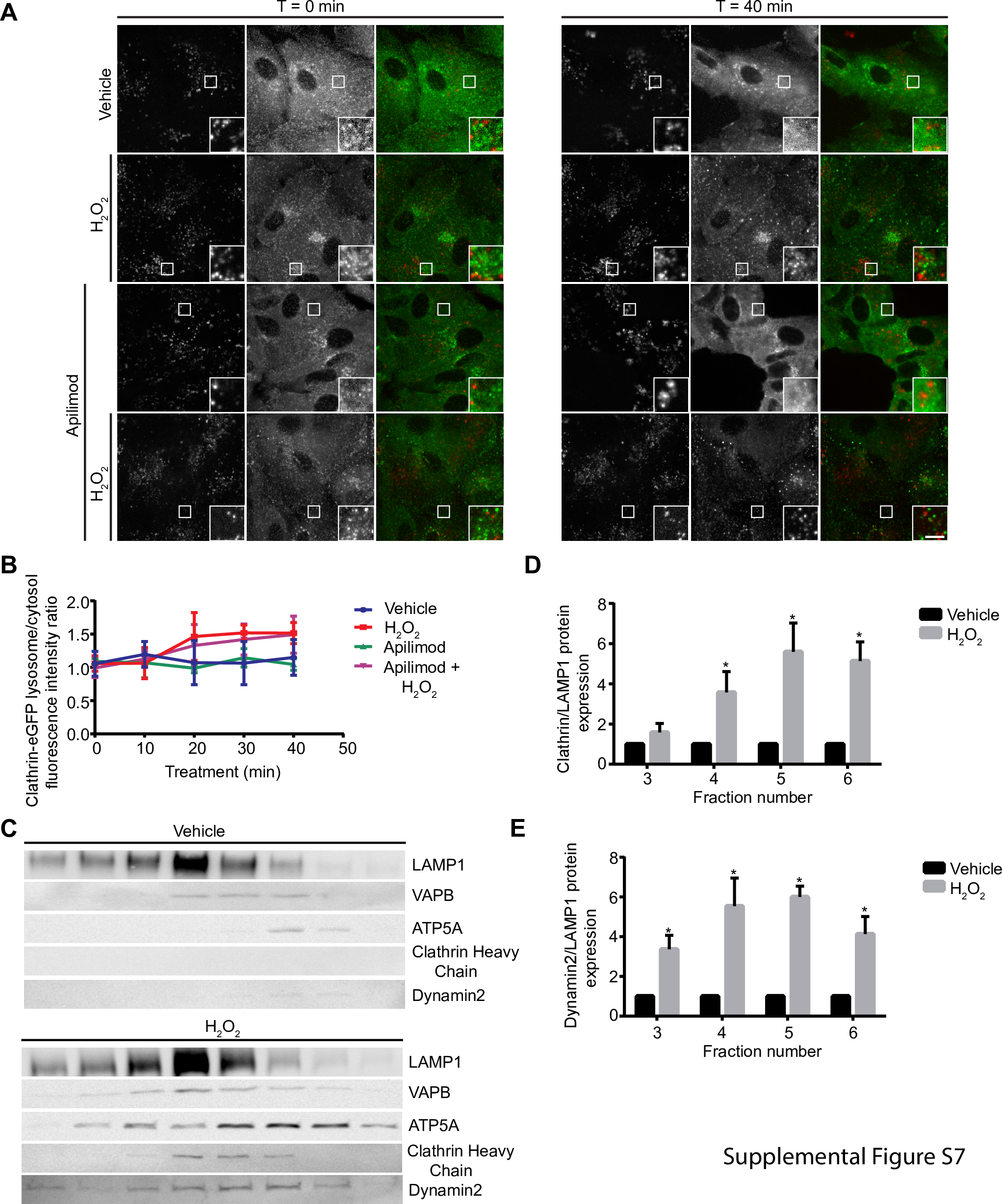

Supplement: S7 Fig — (A) RPE cells stably expressing clathrin heavy chain-eGFP were pre-labelled with Alexa546-conjugated dextran and treated with vehicle, 1 mM H2O2, or 200 nM apilimod with or without 1 mM H2O2. Single z- plane images were acquired every 2 min for 40 min across all treatments. Fluorescence micrographs represent single z-plane images at 0 min and 40 min for each treatment obtained by spinning disc microscopy. The inset is a magnified portion of field of view tracking Alexa546-conjugated dextran lysosome(s) or clathrin-eGFP separate or merged. Scale bar: 7 μm. B. Ratio of clathrin-eGFP fluorescence intensities associated with Alexa546-conjugated dextran and cytosol time points: 0, 10, 20, 30, and 40 min. Data are represented as mean ± s.e.m. from five to six independent experiments, with 1–3 cells assessed per treatment condition per experiment. Two-way ANOVA and Tukey’s post-hoc test were used for (B), where * indicates P<0.05 against control conditions. (C) RAW cells were treated with vehicle or 1 mM H2O2 for 40 min, lysed and homogenates fractionated through a sucrose gradient ultracentrifugation. Fractions were immunoblotted against LAMP1 and VAPB to respectively identify lysosome and ER fractions, and aganst clathrin heavy chain and dynamin 2. Protein expression for clathrin heavy chain (D) or dynamin 2 (E) were normalized to LAMP1 for fractions 3 to 6. Data are represented as mean ± s.d. from three independent experiments. Unpaired Student’s t-test was used for (D-E), where * indicates P<0.05 against vehicle control conditions. (TIF) [file pone.0259313.s007.tif]

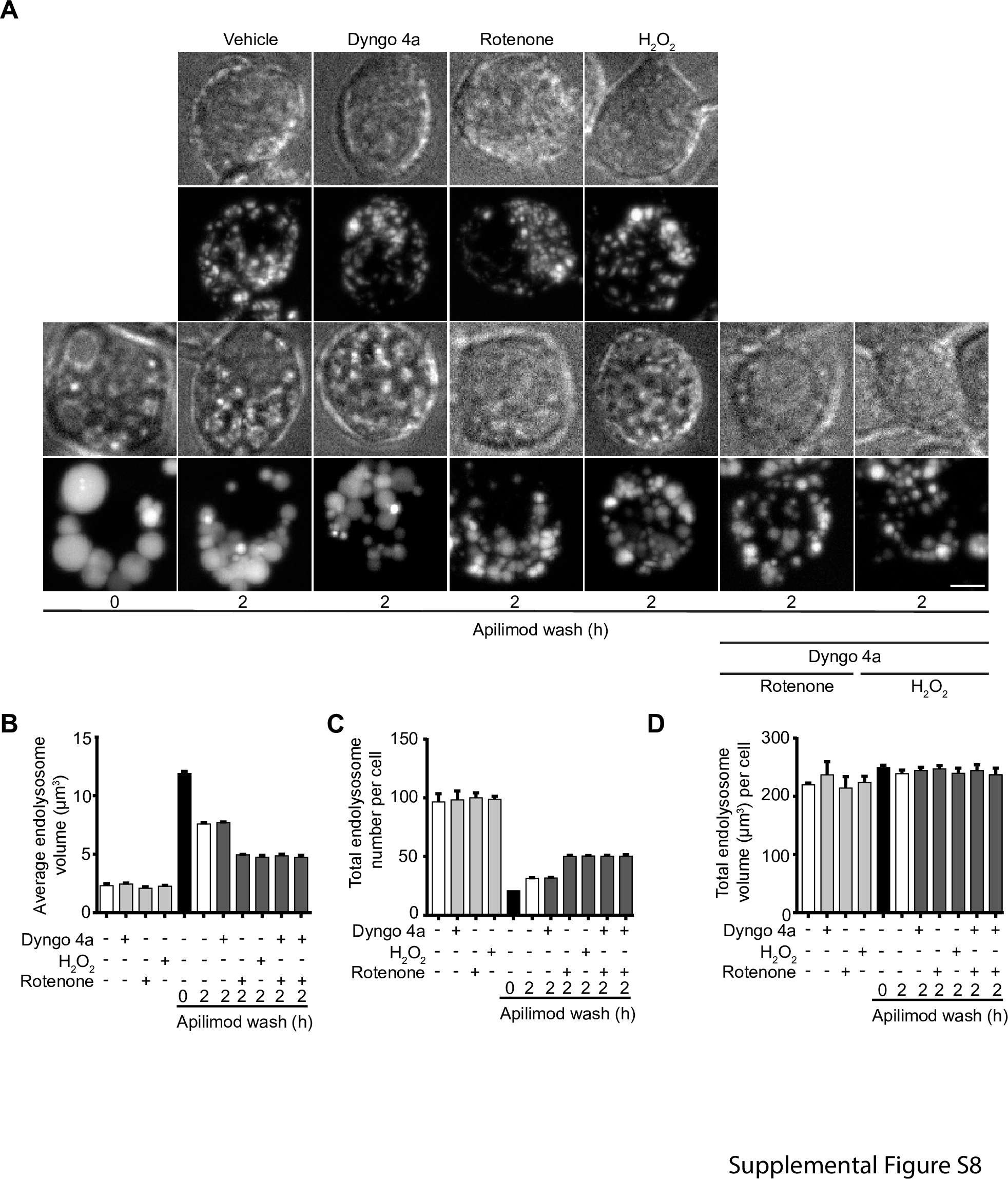

Supplement: S8 Fig — (A) RAW cells were pre-labelled with Lucifer yellow and exposed to either vehicle, 30 μM dyngo-4A for 2 h, 1 mM H2O2 for 40 min, or 1 μM rotenone for 1 h, or 20 nM apilimod for 60 min. Additional subgroup of apilimod treated cells were then washed and incubated with apilimod-free media and changed for 2 h in the presence of vehicle, dyngo-4A, H2O2, and dyngo4-A plus H2O2 for a total time of 2 h without apilimod. Fluorescence micrographs are spinning disc microscopy images with 45–55 z-planes represented as z-projections. Scale bar: 5 μm. (B-D) Quantification of individual lysosome volume (B), lysosome number per cell (C), and total lysosome volume per cell (D). Data is illustrated as mean ± s.e.m. from three independent experiments, with 25–30 cell assessed per treatment condition per experiment. One-way ANOVA and Tukey’s post-hoc test used for B-D with *P<0.05 compared to indicated control conditions. (TIF) [file pone.0259313.s008.tif]
