# Supplementary material for: Reactive oxygen species prevent lysosome coalescence during PIKfyve inhibition
Source: PLoS One. 2021 Nov 23;16(11):e0259313. doi: 10.1371/journal.pone.0259313 (PMC8610251; doi:10.1371/journal.pone.0259313)

Western Blot Images

Cytosolic Materials

1) Apilimod
2) Apilimod+Rotenone
3) Apilimod+H_2O_2
4) Vehicle
5) Rotenone
6) H_2O_2

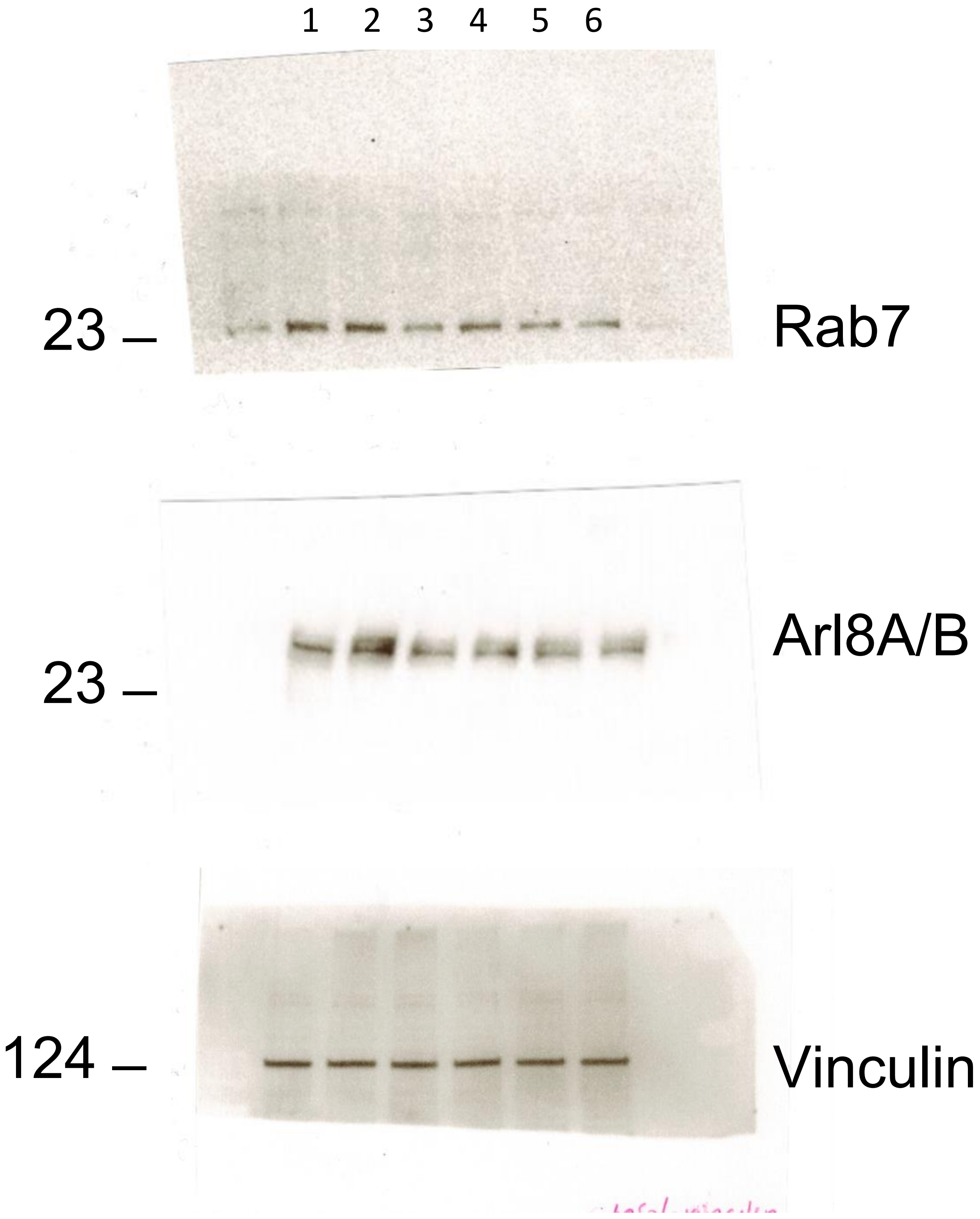

Western Blot Images
Membrane Materials

1) Apilimod
2) Apilimod+Rotenone
3) Apilimod+H_2O_2
4) Vehicle
5) Rotenone
6) H_2O_2

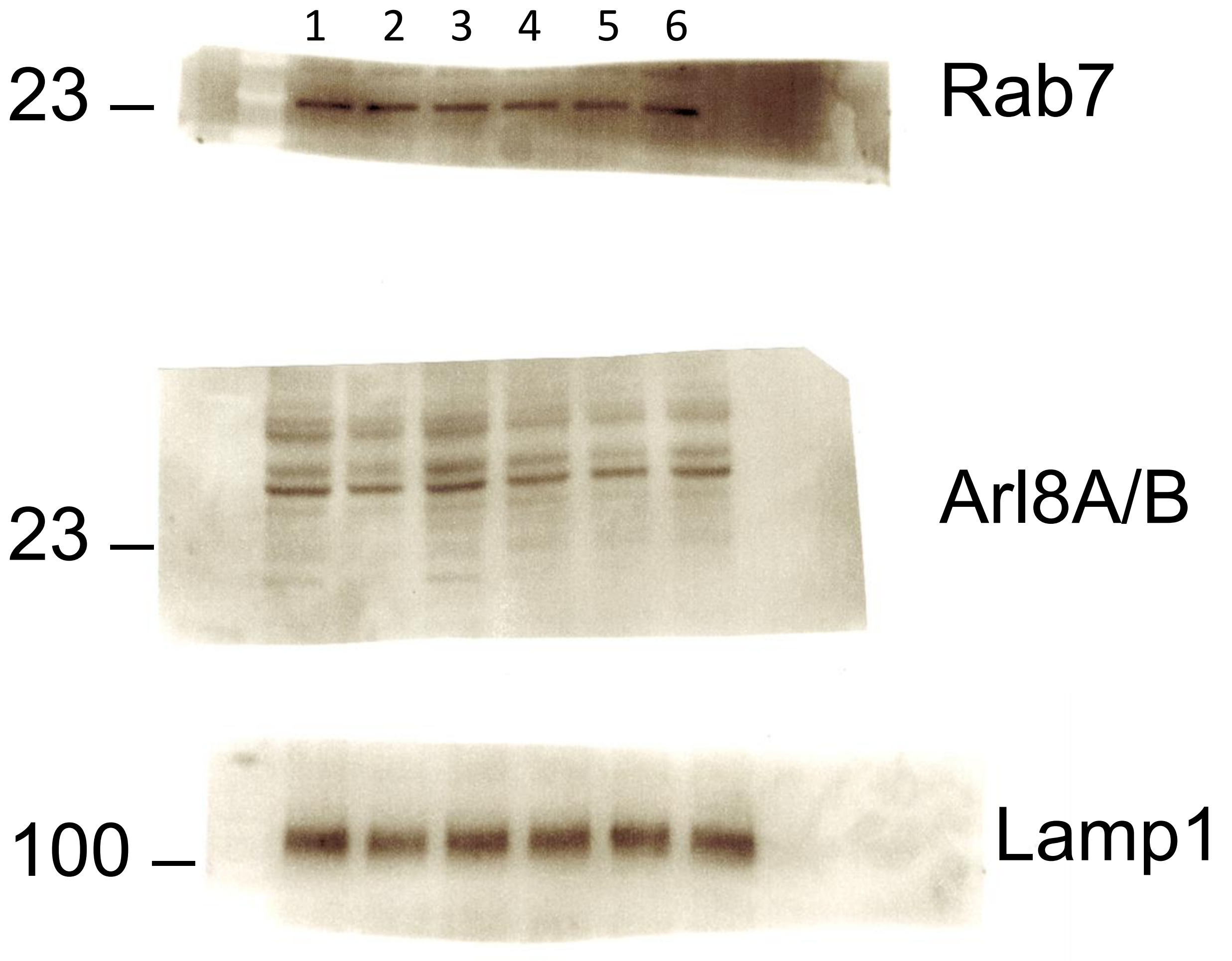

Lysosome fractionation western blot

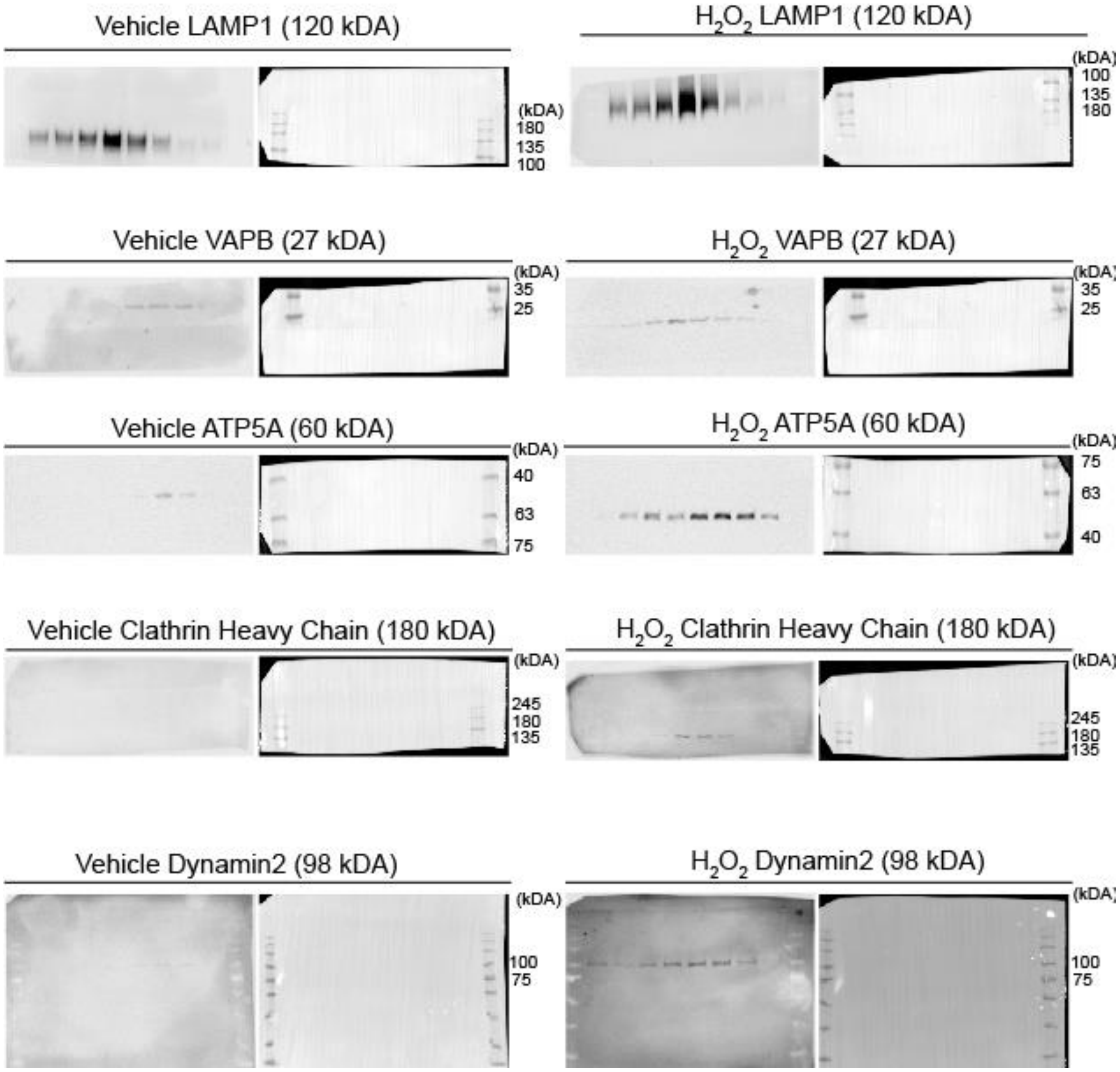

Supplement: S1 Raw images — (PDF) [file pone.0259313.s009.pdf]
