# Supplementary material for: Reactive oxygen species prevent lysosome coalescence during PIKfyve inhibition
Source: PLoS One. 2021 Nov 23;16(11):e0259313. doi: 10.1371/journal.pone.0259313 (PMC8610251; doi:10.1371/journal.pone.0259313)
Supplement: Attachment — Submitted filename: rebuttal plan 20210616.docx [file pone.0259313.s026.docx]

**Response to Peer Review – Saffi et al.**

**Reviewer 1**

We thank the Reviewer for the very positive outlook on our manuscript and work. We addressed their comment below as follows.

*#1.1: “..I have only a minor comment. The authors show that ROS accumulation subside the Apilimod-mediated lysosomal coalescence. Does ROS accumulation also prevent the lysosomal degradative activity impaired by PIKfyve inhibition?”*

ANSWER: While there are reports that PIKfyve inhibition can impair lysosome degradative activity, this is not without some controversy. In fact, we tried to address this point by using the Magic Red Cathepsin substrate, a fluorogenic probe that measures Cathepsin B activity, whereby increased fluorescence indicates enhanced degradation. To account for differences in loading, we normalized fluorescence of Magic Red to Alexa488-conjugated dextran fluorescence pre-loaded onto lysosomes. We then measured the ratio of Magic Red to Alexa488-conjugated dextran per cell in resting cells vs. apilimod-treated cells. However, within the time frame of apilimod treatment that is relevant to lysosome coalescence and prevention by ROS (1-1.5 h), we did not observe a significant inhibition of Magic Red fluorescence relative to vehicle condition. Thus, there was no sufficient inhibition of degradative capacity in apilimod-treated cells to carry out the suggested experiment. We did include measurement of Magic Red fluorescence in cells also treated with H2O2 or rotenone. While we didn’t see a significant effect by rotenone, H2O2 appeared to reduce this activity. Thus, if anything, H2O2 impairs lysosome degradative capacity despite preventing apilimod-induced lysosome coalescence. We include the data here but opted not to include this in the revised manuscript since we believe it detracts from the main focus on the paper. If the Reviewer feels strongly, we can include it, but we would prefer not to.

**Reviewer 2**

We would like to thank the Reviewer #2 for their assessment and for raising the technical issues that we addressed below:

#2.1: *“I have few technical concerns. One is that the concentrations of H2O2 used are extremely high (even the 0.5mM is high, and sometimes this has no effect; 5mM is excessively high), much higher than would be observed in cells, so it is hard to see how this can be minimally connected with what happens in living cells.”*

ANSWER: We appreciate this comment and agree with the sentiment. As shown in the new S1 Supplemental Figure, 100 µM H_2_O_2_ prevent lysosome coalescence caused by 1 or 5 nM apilimod. We reduced the apilimod concentration to do these experiments in case 20 nM apilimod “overpowered” the lower 100 µM H_2_O_2_. We mention this starting in line 138.

*#2.2: The second is that acute exposure to ROS has already been shown to promote MCOLN1 activity, which would explain the phenotypes observed.*

ANSWER: We believe the Reviewer 2 is referring to the study by Zhang et al. (2016, Nat. Comm). In this work, the authors did not examine lysosome morphology, which is the key parameter of our observations. We also would argue that MCOLN1 is not likely to be the only effector of PIKfyve and PtdIns(3,5)P_2_ and thus, we caution about extrapolating MCOLN1 activity to all aspects of lysosome dynamics/morphology. Regardless, we decided to determine if Ca^2+^ has a role in how ROS prevent lysosome enlargement in PIKfyve-inhibited cells. First, we estimated the relative Ca^2+^ levels in lysosomes and cytosol using Fluo4-AM, and second, we tested whether Ca^2+^ chelation with BAPTA-AM would neutralize the ROS effect on apilimod-induced lysosome coalescence.

Consistent with H_2_O_2_-mediated activation of MCOLN1, we did observe an increase in cytosolic Ca^2+^ relative to lysosome (Figure 10). However, this was not observed for rotenone, suggesting that not all ROS have this effect on Ca^2+^ release from lysosomes (Figure 10). Moreover, when we treated cells with BAPTA-AM to chelate Ca^2+^, we did not observe any effect on basal lysosome morphology, apilimod-induced lysosome enlargement, or ROS-prevention of this lysosome enlargement (Figure 10). Overall, we do not think that Ca^2+^ (and MCOLN1) are implicated in how ROS prevents lysosome enlargement during PIKfyve inhibition. This is not to say that ROS-MCOLN1 do not play a role in regulating other aspects of lysosome dynamics, as described in Zhang et al. This is described starting in line 280.

**Reviewer 3**

We appreciate and thank Reviewer #3 for their comments and suggestions. We have addressed these fully as described below.

*#3.1: “Title: using “rescue” indicate a protective effect but I don’t see any evidence that ROS actually rescue lysosome function and cause betterment of the cell. In my opinion, using a functional assay such as lysotracker is needed be to show this. However, I would expect that ROS induction will also be deteriorative for lysosomal function and therefore large lysosomes or multiple small ones will not make a difference for the cell, unless macrophages are an exception.”*

ANSWER: We replaced “rescue” in the title to the more appropriate “prevent” to address this concern.

*#3.2 “The short title reads like a review article and should be changed.”*

ANSWER: We changed the short title to “Reactive oxygen species prevent lysosome enlargement” to be more descriptive of the work.

*#3.3: “Throughout the introduction, some detailed explanations in relation to cargo delivery or lysosomal movement can be summarized or deleted. Also none of the abbreviations have been described and there are too many of them.”*

ANSWER: We simplified the introduction and defined additional gene names.

*#3.4: “The inhibitors specificity: CDNB is not a specific inhibitor of TrxR1. It is generally used to quench all thiols. Auranofin would be a more appropriate inhibitor of TrxR1, or perhaps PX12 for inhibition of Trx can be another specific inhibitor.”*

ANSWER: To address this important point, we used auranofin in experiments inducing lysosome enlargement with apilimod. We observed that auranofin impaired lysosome coalescence induced by apilimod – this is shown in Figure 2. Given that this result is similar to other reagents, we thought it was reasonable to not repeat other experiments with auranofin.

*#3.5: “While the effect of these inhibitors on cellular skeleton was shown elegantly, I did not see specific assay to identify specific types of ROS. Since this is a major concept in this study that different ROS have different effects, authors need to specify the type of ROS that is generated during these conditions for their conclusions. Using commercially available kits (such as mitosox) is required.”*

ANSWER: Thank you for the suggestion. We obtained HPF as a probe for hydroxyl radical/peroxynitrite, ROS-ID as a probe for superoxide, MitoSox as a probe for mitochondrial superoxide, and si-DMA as an indicator for singlet O2. We observed that H2O2 produced all ROS except singlet O2; we expect H2O2 generates these ROS throughout the cytoplasm. In comparison, rotenone produced mitochondrial superoxide, as expected, but also singlet O2. CDNB also produced singlet O2. We didn’t observe a signal with MCB though we detected ROS by CellRox and GSH-MCB adduct formation. This is now shown in the new Figure 1 and described in text, starting in line 123. Collectively, we propose in the discussion, starting in line 401, that H2O2 itself, and possibly the global production of superoxide and hydroxyl radical, stabilizes microtubules and impairs motor activity. In turn, mitochondrial superoxide and/or singlet O2 may destabilize microtubules and actin, including actin on lysosomes.

*3.6: “An obvious question regarding ROS impact on lysosome has been neglected: ROS are known to induce lipid peroxidation and lysosomal membrane permeabilization (LMP). Is this “rescue effect” mediated because of membrane damage? Lipid peroxidation can be readily assessed by commercial kits. Also using cell fractionation and variety of lysosomal probes that the group has, the involvement of LMP can be quantified.”*

To test whether ROS damaged lysosomes, we expressed Galectin-3-GFP. Under the conditions we used (1 h or so), we did not observe a significant increase in galectin-3 positive structures in ROS-treated cells and thus, we do not think that lysosome damage explains how ROS broadly prevent lysosome enlargement during PIKfyve inhibition. This is shown in Figure 9 and presented starting in line 271.

*#3.7: “Some factors such as GTPases that have been quantified by signal intensity must be quantified by western blotting.”*

We fractionated cells into membrane and cytosolic pools after treatment with apilimod with or without ROS induction (H2O2, rotenone). As with imaging, we did not observe a significant shift in membrane-bound Rab7 or Arl8b between these conditions. This is found in S5 Supplemental Figure and indicated in line 246.

*#3.8: “If possible, simplifying the language will also make it easier to read.”*

ANSWER: We shortened the introduction, which was on the long side.
